# Polyploidization enhances plant resistance to *Alternaria alternata* via DNA hypomethylation activated WRKYs

**DOI:** 10.1093/hr/uhag050

**Published:** 2026-02-19

**Authors:** Zhongyu Yu, Huiting Ci, Ruyue Jing, Qi Yu, Jun He, Ye Liu, Jiafu Jiang, Haibing Wang, Weimin Fang, Zhenxing Wang, Fadi Chen

**Affiliations:** State Key Laboratory of Crop Genetics & Germplasm Enhancement and Utilization, Key Laboratory of Flower Biology and Germplasm Innovation, Ministry of Agriculture and Rural Affairs, Key Laboratory of State Forestry and Grassland Administration on Biology of Ornamental Plants in East China, College of Horticulture, Nanjing Agricultural University, Nanjing, Jiangsu 211800, China; Zhongshan Biological Breeding Laboratory, No.50 Zhongling St, Nanjing, Jiangsu 210014, China; State Key Laboratory of Crop Genetics & Germplasm Enhancement and Utilization, Key Laboratory of Flower Biology and Germplasm Innovation, Ministry of Agriculture and Rural Affairs, Key Laboratory of State Forestry and Grassland Administration on Biology of Ornamental Plants in East China, College of Horticulture, Nanjing Agricultural University, Nanjing, Jiangsu 211800, China; Zhongshan Biological Breeding Laboratory, No.50 Zhongling St, Nanjing, Jiangsu 210014, China; State Key Laboratory of Crop Genetics & Germplasm Enhancement and Utilization, Key Laboratory of Flower Biology and Germplasm Innovation, Ministry of Agriculture and Rural Affairs, Key Laboratory of State Forestry and Grassland Administration on Biology of Ornamental Plants in East China, College of Horticulture, Nanjing Agricultural University, Nanjing, Jiangsu 211800, China; Zhongshan Biological Breeding Laboratory, No.50 Zhongling St, Nanjing, Jiangsu 210014, China; State Key Laboratory of Crop Genetics & Germplasm Enhancement and Utilization, Key Laboratory of Flower Biology and Germplasm Innovation, Ministry of Agriculture and Rural Affairs, Key Laboratory of State Forestry and Grassland Administration on Biology of Ornamental Plants in East China, College of Horticulture, Nanjing Agricultural University, Nanjing, Jiangsu 211800, China; Zhongshan Biological Breeding Laboratory, No.50 Zhongling St, Nanjing, Jiangsu 210014, China; State Key Laboratory of Crop Genetics & Germplasm Enhancement and Utilization, Key Laboratory of Flower Biology and Germplasm Innovation, Ministry of Agriculture and Rural Affairs, Key Laboratory of State Forestry and Grassland Administration on Biology of Ornamental Plants in East China, College of Horticulture, Nanjing Agricultural University, Nanjing, Jiangsu 211800, China; Zhongshan Biological Breeding Laboratory, No.50 Zhongling St, Nanjing, Jiangsu 210014, China; State Key Laboratory of Crop Genetics & Germplasm Enhancement and Utilization, Key Laboratory of Flower Biology and Germplasm Innovation, Ministry of Agriculture and Rural Affairs, Key Laboratory of State Forestry and Grassland Administration on Biology of Ornamental Plants in East China, College of Horticulture, Nanjing Agricultural University, Nanjing, Jiangsu 211800, China; Zhongshan Biological Breeding Laboratory, No.50 Zhongling St, Nanjing, Jiangsu 210014, China; State Key Laboratory of Crop Genetics & Germplasm Enhancement and Utilization, Key Laboratory of Flower Biology and Germplasm Innovation, Ministry of Agriculture and Rural Affairs, Key Laboratory of State Forestry and Grassland Administration on Biology of Ornamental Plants in East China, College of Horticulture, Nanjing Agricultural University, Nanjing, Jiangsu 211800, China; Zhongshan Biological Breeding Laboratory, No.50 Zhongling St, Nanjing, Jiangsu 210014, China; State Key Laboratory of Crop Genetics & Germplasm Enhancement and Utilization, Key Laboratory of Flower Biology and Germplasm Innovation, Ministry of Agriculture and Rural Affairs, Key Laboratory of State Forestry and Grassland Administration on Biology of Ornamental Plants in East China, College of Horticulture, Nanjing Agricultural University, Nanjing, Jiangsu 211800, China; Zhongshan Biological Breeding Laboratory, No.50 Zhongling St, Nanjing, Jiangsu 210014, China; State Key Laboratory of Crop Genetics & Germplasm Enhancement and Utilization, Key Laboratory of Flower Biology and Germplasm Innovation, Ministry of Agriculture and Rural Affairs, Key Laboratory of State Forestry and Grassland Administration on Biology of Ornamental Plants in East China, College of Horticulture, Nanjing Agricultural University, Nanjing, Jiangsu 211800, China; Zhongshan Biological Breeding Laboratory, No.50 Zhongling St, Nanjing, Jiangsu 210014, China; State Key Laboratory of Crop Genetics & Germplasm Enhancement and Utilization, Key Laboratory of Flower Biology and Germplasm Innovation, Ministry of Agriculture and Rural Affairs, Key Laboratory of State Forestry and Grassland Administration on Biology of Ornamental Plants in East China, College of Horticulture, Nanjing Agricultural University, Nanjing, Jiangsu 211800, China; Zhongshan Biological Breeding Laboratory, No.50 Zhongling St, Nanjing, Jiangsu 210014, China; State Key Laboratory of Crop Genetics & Germplasm Enhancement and Utilization, Key Laboratory of Flower Biology and Germplasm Innovation, Ministry of Agriculture and Rural Affairs, Key Laboratory of State Forestry and Grassland Administration on Biology of Ornamental Plants in East China, College of Horticulture, Nanjing Agricultural University, Nanjing, Jiangsu 211800, China; Zhongshan Biological Breeding Laboratory, No.50 Zhongling St, Nanjing, Jiangsu 210014, China

## Abstract

Polyploidization is a major driver of plant evolution and stress adaptation, yet its role in modulating biotic stress resistance through epigenetic mechanisms remains poorly understood. This study demonstrates that autotetraploidization in *Chrysanthemum lavandulifolium* significantly enhances resistance to *Alternaria alternata*, the cause of black spot disease. Whole-genome methylome and transcriptome analyses reveal that polyploidization induces locus-specific CHH hypomethylation in the promoters of a subset of WRKY transcription factors, leading to their transcriptional activation upon fungal infection. Functional characterization of *CIWRKY103*, a key hypomethylated WRKY gene, confirms its critical role in conferring disease resistance. Chemical inhibition of DNA methylation (5-azacytidine treatment) in diploid plants mimics the tetraploid phenotype by activating *WRKY103* expression and enhancing resistance. This epigenetic regulatory mechanism is conserved across diverse chrysanthemum species, highlighting the potential of targeting DNA methylation to modulate fungal disease resistance in polyploid crops. Our findings unveil a novel link between polyploidy, epigenetic reprogramming, and pathogen defense, offering strategic insights for sustainable crop protection.

## Introduction

Whole-genome duplication (WGD), a cornerstone mechanism driving plant diversification and adaptation, serves as a fundamental catalyst for genomic novelty and functional innovation [1]. Compared with diploids, polyploids generally exhibit increased adaptability to both biotic and abiotic stresses including salt, cold, heat, fungal disease, etc. in plants [2–9]. After genome duplication in plants, dynamic genomic changes are often accompanied by alterations in epigenetic patterns. Research reveals that DNA methylation, as a key epigenetic regulator, plays a central role in maintaining genome stability and regulating environmental adaptability in polyploids [10]. In *Arabidopsis*, DNA methylation is considered one of the contributors that may improve genome stability and adaptation during polyploid evolution [11]. In autotetraploid *Brassica rapa*, the DNA methylation levels near genes are negatively correlated with transcriptional activity, indicating that DNA methylation effectively regulates gene expression in polyploids to counteract genome shock [12]. Compared with diploids, autotetraploid *Oryza sativa* were more tolerant to salt stress due to DNA hypomethylated regions enriched for jasmonic acid (JA) biosynthesis- and signaling-related genes and these genes were activated earlier and more intensely under salt stress [13]. Autopolyploidization improved salt tolerance in *Citrus reticulata* via DNA hypomethylation, which activated the ethylene biosynthesis pathway [3]. Current reported work has revealed the connection between epigenetic modifications and tolerance to abiotic stresses during polyploidization, whereas epigenetic regulation of polyploids in response to biotic stresses is lacking in-depth research.

*Alternaria* Nees are necrotrophic pathogenic fungi capable of infecting a wide variety of economically and ecologically important crops and pose severe threats to agricultural production worldwide. Therefore, elucidating plant defense response mechanisms to *Alternaria* Nees-induced diseases have become key research priorities. In *Arabidopsis thaliana*, *B. oleracea*, *Nicotiana attenuata*, and *Chrysanthemum morifolium*, transcription factors (TFs) such as WRKYs and ERFs could integrate hormone signals to activate the biosynthesis of phytoalexins such as capsidiol and indolic glucosinolates, thereby synergistically improving resistance against *Alternaria alternata* [14–17]. DNA methylation, as an epigenetic modification, regulates TFs, such as WRKYs, in response to abiotic stresses in plants [18–20]. However, whether the enhanced resistance to *A. alternata* in polyploid plants is associated with DNA methylation-regulated TFs such as WRKYs remains unclear.

*Chrysanthemum*, a typical Asteraceae genus, is economically important in ornamental, pharmaceutical, and beverage industries. Its suffering by *A. alternata-*induced black spot disease (BSD) represents a common disease that causes industrial loss in lots of economic crops. The diploid species *C. lavandulifolium* (2*n* = 2 × =18) is considered a key ancestral progenitor of cultivated *Chrysanthemum*. In this study, we found that, compared with diploids, autotetraploid *C. lavandulifolium* (2*n* = 4 × =36) presented significantly greater resistance to BSD. Whole-genome methylation analysis revealed that a locus-specific reduction in CHH (H represents A, T or C) methylation targeted a group of WRKY promoters after *A. alternata* inoculation in autotetraploid *C. lavandulifolium*. The reduced CHH methylation led to strong transcriptional activation of these targeted WRKY genes, of which *ClWRKY103* was the strongest in autotetraploids. *ClWRKY103* overexpression significantly improved resistance to BSD in diploid *C. lavandulifolium*. Treatment of diploid *C. lavandulifolium* with the DNA methylation inhibitor 5-azacytidine (5-AzaC) led to CHH hypomethylation at the *ClWRKY103* promoter, which resulted in the activation of *ClWRKY103* expression and increased BSD resistance. Autopolyploidization in another wild diploid, *C. indicum* (Shennongjia, Hubei), and 5-AzaC treatment in *C. indicum* and the cultivar *C. morifolium* ‘Jinba’ resembled similar phenotype in *C. lavandulifolium.* Taken together, the results demonstrated that enhanced resistance to *A. alternata* in autotetraploids caused by CHH hypomethylation-activated WRKYs could be considered a common mechanism to address the threat of *A. alternata* through controlling epigenetic modifications in various chrysanthemums and probably other plant species.

## Results

### Polyploidization enhanced resistance to *A. alternata* in *C. lavandulifolium*

**Figure 1:**
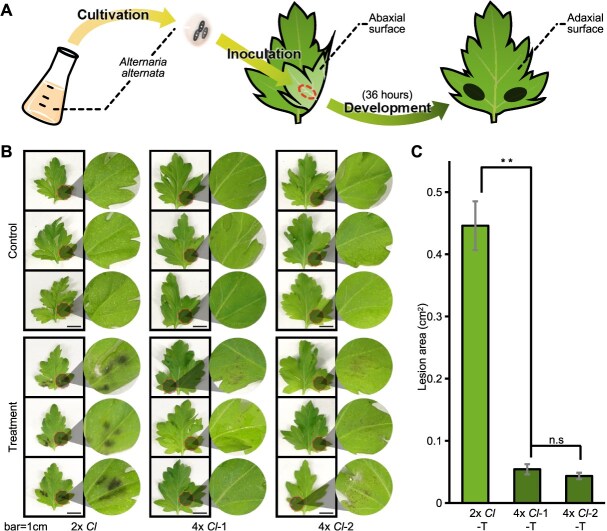
Compared with diploids, autotetraploid *C. lavandulifolium* presented increased resistance to *A. alternata*. **(A)** Schematic of the inoculation procedure with *A. alternata.*  **(B)** Phenotypic response of symptomatic leaves to *A. alternata* inoculation. 2x *Cl*, diploid *C. lavandulifolium*; 4x *Cl*-1, autotetraploid *C. lavandulifolium* line 1; 4x *Cl*-2, autotetraploid *C. lavandulifolium* line 2. Scale bar: 1 cm. **(C)** Statistical analysis of leaf disease incidence. T: Treatment group inoculated with *A. alternata*. Error bars represent the standard error (SE, *n* = 10). Significance of differences: **P < 0.01, n.s: not significant (one-way ANOVA). Leaf disease incidence: calculated as the ratio of lesion area to total leaf area, quantified by ImageJ.

To test the effect of polyploidization in response to BSD, we inoculated diploid *C. lavandulifolium* (2*x Cl*, Fig. S1) and two autotetraploid *C. lavandulifolium* lines (4*x Cl*-1 and -2, Fig. S1) obtained via colchicine treatment via mycelial balls of *A. alternata*. After 36 h of incubation under long-day conditions, the disease incidence of the leaves on the obverse side was recorded (Fig. 1A). The results revealed that 4*x Cl* significantly enhanced disease resistance following *in planta* inoculation with *A. alternata* (Fig. 1B). Quantitative analysis of leaf disease incidence via ImageJ software revealed a significant reduction (*P* < 0.01) in necrotic lesion development on autotetraploid leaves compared with their diploid counterparts (Fig. 1C). These findings suggest that polyploidization is indeed positively associated with resistance to *A. alternata* in *C. lavandulifolium*.

### WRKY genes highly respond to *A. alternata* infection in autotetraploid *C. lavandulifolium*

**Figure 2:**
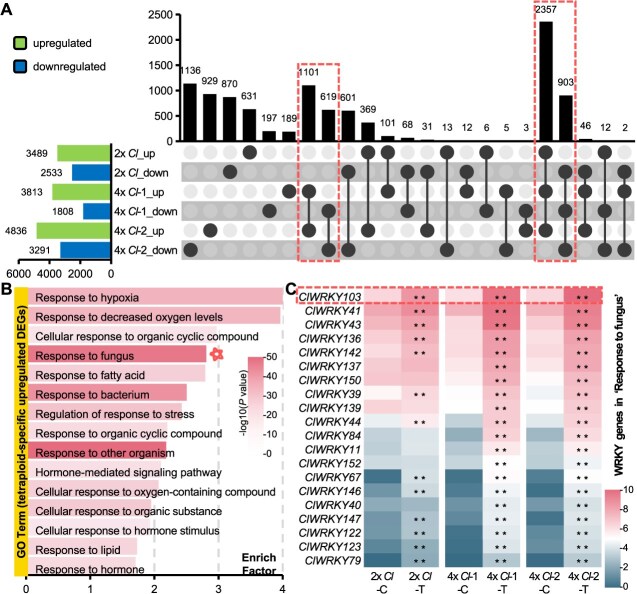
Transcriptional responses of 2x and 4x *C. lavandulifolium* after *A. alternata* treatment. **(A)** UpSet plot of DEGs in 2x and 4x *C. lavandulifolium.* DEGs: |log_2_FoldChange| > 1, *P* < 0.01. 2x *Cl*, diploid *C. lavandulifolium*. 4x *Cl*-1, autotetraploid *C. lavandulifolium* line 1. 4x *Cl*-2, autotetraploid *C. lavandulifolium* line 2. C: Control group. T: Treatment group inoculated with *A. alternata*. Green: upregulated DEGs. Blue: downregulated DEGs. **(B)** GO enrichment analysis of the tetraploid-specific upregulated DEGs. **(C)** Expression patterns of WRKY genes in ‘Response to fungus’. Color bar represents normalized gene expression levels (TPM). Significance of differences: **P < 0.01, log_2_FoldChange > 1.

To explore the transcriptomic response, leaves inoculated with *A. alternata* for 36 h were collected from the plants for RNA-seq (Table S1 and Fig. S2A). After inoculation, genes that were commonly upregulated (treated vs control, log_2_FoldChange > 1, *P* < 0.01) in the 2*x Cl* and two 4*x Cl* lines, with higher transcriptional levels in the two 4*x Cl* lines than in the 2*x Cl* lines (4*x* treated vs 2*x* treated, FoldChange >1.5), and the genes upregulated only in both 4*x Cl* lines were considered tetraploid-specific upregulated genes (Fig. 2A, Table S2). Conversely, tetraploid-specific downregulated genes were defined with similar concepts (Fig. 2A, Table S3). Gene ontology (GO) enrichment analysis revealed the significant term ‘Response to fungus’ (GO:0009620) in the set of tetraploid-specific upregulated genes (Fig. 2B), which is consistent with the increased tolerance to *A. alternata* in autotetraploid *C. lavandulifolium*. However, no terms directly associated with plant disease response were identified from the tetraploid-specific downregulated genes (Fig. S2B). Interestingly, plenty of WRKY genes were deregulated in tetraploid plants in response to *A. alternata.* For comprehensive analysis of WRKYs, 167 WRKY genes were identified in *C. lavandulifolium* (Figs S3 and S4)*.* Further analysis revealed enrichment of WRKYs among the TFs (20/25, 80%) associated with the response of genes to fungus (Figs 2C and S5). These results suggest that WRKYs likely play important roles in regulating the defense of *A. alternata* in autotetraploid *C. lavandulifolium*.

### CHH hypomethylation was associated with high activation of WRKY genes and BSD resistance

**Figure 3:**
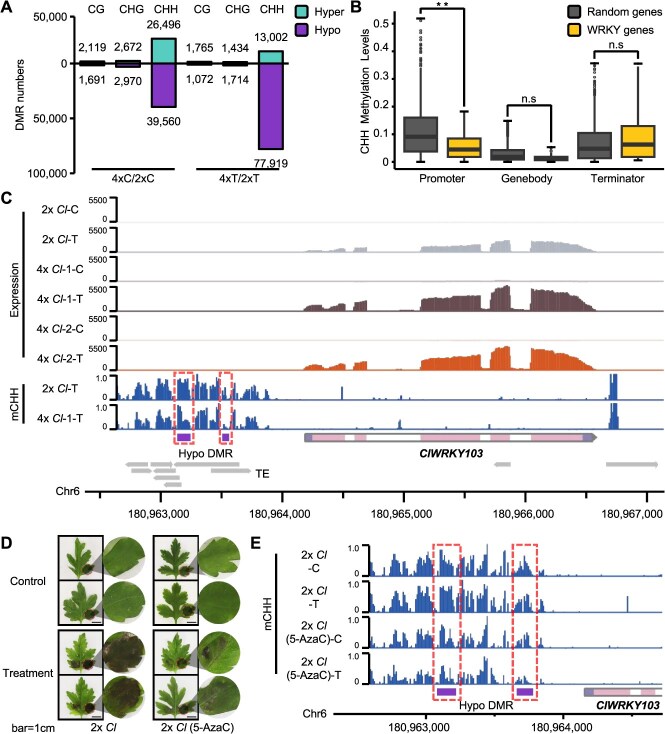
DNA hypomethylation induced the upregulation of *ClWRKY103*. **(A)** DMR number statistics. Cyan: Hypermethylation DMR; Purple: Hypomethylation DMR. **B** Differences in CHH methylation levels. Golden: WRKY genes; gray: 1000 randomly selected genes; **P < 0.01; n.s: not significant (one-way ANOVA). **(C)** Genome browser snapshots showing expression level and CHH methylation changes in the promoter and gene body regions of *ClWRKY103*. Panel Expression displays six tracks of gene expression levels for different samples, with the *y*-axis from 0 to 5500 representing the expression levels in TPM. Panel mCHH displays two tracks of CHH methylation levels for different samples, with the *y*-axis from 0 to 1.0 representing the methylation ratio from 0% to 100%. 2x *Cl*, diploid *C. lavandulifolium*; 4x *Cl*-1, autotetraploid *C. lavandulifolium* line 1; 4x *Cl*-2, autotetraploid *C. lavandulifolium* line 2; C, Control group; T, Treatment group inoculated with *A. alternata*; Pink, Exon; Light purple, Untranslated Region; Purple, Hypomethylated DMR. **(D)** Phenotypic response of 2x *Cl*- and 5-AzaC-treated leaves to *A. alternata* inoculation. Scale bar: 1 cm. **(E)** Genome browser snapshots showing changes in CHH methylation of the *ClWRKY103* promoter region after 5-AzaC treatment. Panel mCHH displays four tracks of CHH methylation levels for different samples, with the *y*-axis from 0 to 1.0 representing the methylation ratio from 0% to 100%.

Polyploidization normally accompanies a reshaped DNA methylation status. To test whether DNA methylation was involved in regulating the deregulated transcriptome, we performed whole-genome bisulfite sequencing (WGBS) using the same materials collected for RNA-seq (Table S4). The results indicated that there were no strong alterations in DNA methylation genome-wide after *A. alternata* infection (Fig. S6A–C). However, considerable numbers of CHH hypo-DMRs (Differentially Methylated Regions) were identified, particularly in the 4*x Cl* treatment compared with the 2*x Cl* treatment after inoculation (Fig. 3A). These CHH hypo-DMRs preferred promoter regions in the genome (Fig. S7A), which implies that they might be able to evoke the transcription of target genes in response to *A. alternata*. Therefore, we tested whether the tetraploid-specific upregulated WRKYs were the targets of these CHH hypo-DMRs. Consistent with this idea, there was a significant overlap between genes with CHH hypo-DMRs and those with tetraploid-specific upregulated WRKYs (Fig. S7B). In support of the notion that activated transcription results from CHH hypomethylation, these WRKY genes indeed had lower CHH methylation over promoter regions (Fig. 3B) and significantly higher transcriptional levels than randomly selected genes did (Fig. S7C). *ClWRKY103* was the most deregulated WRKY gene in response to *A. alternata* in response to 4*x Cl* (Fig. 2C). After infection, CHH methylation levels in its promoter regions were reduced in 4*x Cl* compared with 2*x Cl* (Fig. 3C), whereas CG and CHG methylation remained largely unchanged (Fig. S8), further suggesting the precise regulation of DNA methylation in autotetraploid plants.

To further confirm the role of DNA methylation in regulating BSD resistance in *C. lavandulifolium*, 5-AzaC treatment was performed before *A. alternata* inoculation via the diploid *C. lavandulifolium*. Strikingly, the 5-AzaC-sprayed group presented a significantly lower lesion incidence after *A. alternata* inoculation than the water-sprayed control (Figs 3D and S9A). The upregulation of *ClWRKY103* in 5-AzaC-sprayed plants after *A. alternata* inoculation mimicked the transcriptional change in *ClWRKY103* in 4*x Cl-*infected plants. WGBS revealed that the upregulation of *ClWRKY103* was indeed due to the decrease in CHH at its promoter upon application of 5-AzaC and *A. alternata* (Figs 3E and S9C–D). These results indicate that DNA methylation likely participates in regulating *A. alternata* resistance through WRKYs such as *ClWRKY103* in *C. lavandulifolium*.

### *ClWRKY103* overexpression enhanced BSD resistance in *C. lavandulifolium*

**Figure 4:**
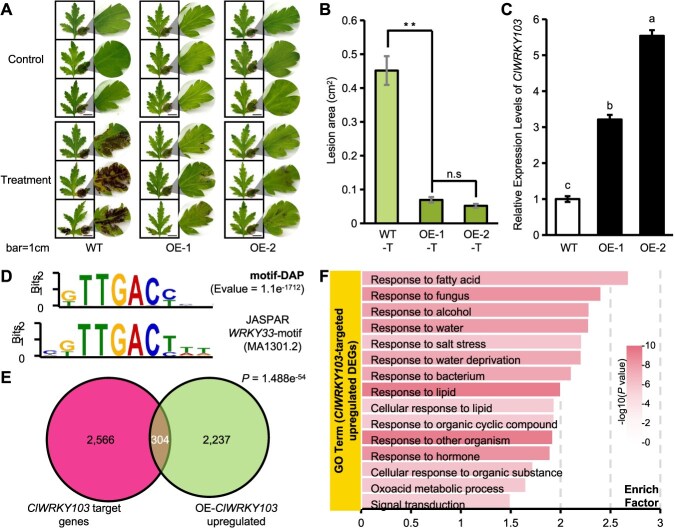
*ClWRKY103* regulates resistance to BSD through downstream genes in *C. lavandulifolium.*  **(A)** Phenotypic response of the leaves of the wild-type (WT) and two *ClWRKY103*-overexpressing lines to *A. alternata* inoculation. Scale bar: 1 cm. **(B)** Statistical analysis of leaf disease incidence. The error bars represent the SE (*n* = 10), and the significance of the differences is as follows: ^**^P < 0.01, n.s: not significant (one-way ANOVA). **(C)** Relative expression levels of *ClWRKY103* in the WT and two *ClWRKY103*-overexpressing lines. Significance of differences: Different lowercase letters indicate significant differences between groups (*P* < 0.01, one-way ANOVA). **(D)** DAP-Seq-derived motif and Arabidopsis *WRKY33* motif reported by JASPAR. The *y*-axis represents the conservation of nucleotides at each position, ranging from 0 to 2, with higher values indicating greater conservation. **(E)** Venn analysis of *ClWRKY103* target genes and upregulated DEGs in OE-*ClWRKY103.* Significance of differences: Hypergeometric test. **(F)** GO enrichment analysis of the upregulated DEGs among the *ClWRKY103*-targeted genes.

To address how the tetraploid-specific upregulated WRKYs function in response to *A. alternata*, we used *ClWRKY103* as an example for subsequent experiments. The overexpression of *ClWRKY103* significantly improved *A. alternata resistance* to 2*x Cl* (Fig. 4A–C), which resembled the phenotype associated with 4*x Cl* with a high abundance of *ClWRKY103*. The results confirmed that *ClWRKY103* is a key factor regulating the response of *C. lavandulifolium* to BSD.

To decipher the downstream elements of ClWRKY103, DNA affinity purification and high-throughput sequencing (DAP-seq) were performed (Table S5). The analysis revealed a highly reliable motif (*E*-value = 1.1e^−1712^), and the identified motif was highly similar to the motif of Arabidopsis WRKY33 reported in JASPAR (Fig. 4D) [21]. We found a significant overlap of genes that were targeted by ClWRKY103 (Table S6) and genes upregulated in *ClWRKY103*-overexpressing plants (Fig. 4E and Table S7). GO analysis revealed that the overlapping genes were strongly enriched for response to fungus and response to bacterium (Fig. 4F). There was also a significant overlap between ClWRKY103 target genes and downregulated genes in *ClWRKY103*-overexpressing plants (Fig. S10A and Table S7). These overlapping genes were highly enriched for photosynthesis (Fig. S10B), which was similar to the findings for the tetraploid-specific downregulated genes. Analysis of the promoter regions of ClWRKY103 targets and *ClWRKY103*-overexpressing upregulated genes enriched in defense [22–24] revealed that these promoters typically harbor multiple *ClWRKY103* motifs (Fig. S10C). The presence of these repeated motifs may enable precise regulatory control by *ClWRKY103*, thereby modulating BSD resistance in *C. lavandulifolium*. Consistently, dual luciferase reporter assays demonstrated that *ClWRKY103* directly associates with the promoters of the downstream genes *ClNPR1* and *ClPDF1.4*, providing functional evidence for its role in transcriptional regulation (Fig. S10D–G).

### Increased *A. alternata* resistance caused by the upregulation of *WRKY103* via hypomethylation might be common in *Chrysanthemum* species

**Figure 5:**
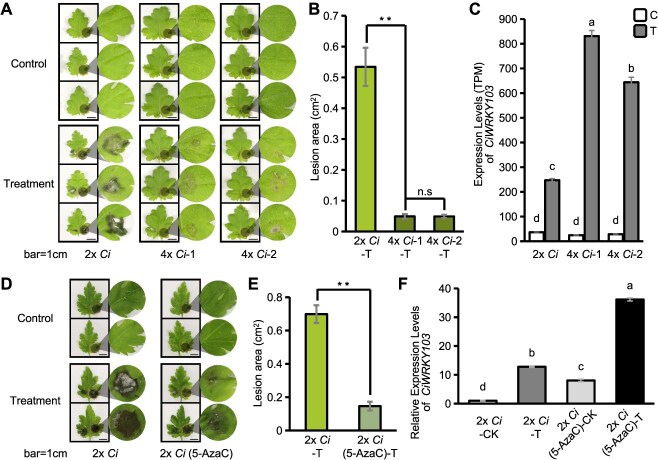
*WRKY103* transcription is associated with BSD resistance in *C. indicum*. **(A)** Phenotypic response of the leaves of diploid *C. indicum* and two autotetraploid *C. indicum* lines to *A. alternata* inoculation. 2x *Ci*, diploid *C. indicum*; 4x *Ci*-1, autotetraploid *C. indicum* line 1; 4x *Ci*-2, autotetraploid *C. indicum* line 2. Scale bar: 1 cm. **(B)** Statistical analysis of leaf disease incidence. The error bars represent the SE (*n* = 10), and the significance of the differences is as follows: **P < 0.01, n.s: not significant (one-way ANOVA). **(C)** Expression levels of *CiWRKY103* in diploid *C. indicum* and two autotetraploid *C. indicum* lines. Significance of differences: Different lowercase letters indicate significant differences between groups (*P* < 0.01, one-way ANOVA). **(D)** Phenotypic response of 2x *Ci-* and 5-AzaC-treated leaves to *A. alternata* inoculation. Scale bar: 1 cm. **(E)** Statistical analysis of leaf disease incidence. Significance of differences: **P < 0.01 (one-way ANOVA). **(F)** Relative expression levels of *CiWRKY103* in the control group versus the *A. alternata-*inoculated treatment group under 2x *Ci* and 2x *Ci* (5-AzaC) conditions. Significance of differences: Different lowercase letters indicate significant differences between groups (*P* < 0.01, one-way ANOVA).

To investigate whether DNA methylation-regulated *WRKY103* plays a role in resistance to BSD in a broader spectrum of *Chrysanthemum* species, we further tested another synthesized autotetraploid chrysanthemum, 4*x C. indicum* (Shennongjia, Hubei) (Fig. S11), and the cultivar *C. morifolium* 'Jinba'. After *A. alternata* inoculation, the 4*x Ci* resembled the enhanced tolerance of 4*x Cl* (Fig. 5A). Compared with 2*x Ci*, both lines of 4*x Ci* presented significantly reduced leaf disease incidence following *A. alternata* inoculation (Fig. 5B). Like in 4*x Cl*, *CiWRKY103* presented remarkably high transcript levels in 4*x Ci* in response to BSD (Fig. 5C). We further tested the role of DNA methylation in the defense against BSD in *C. indicum* via 5-AzaC treatment. The results resembled the resistant phenotype of *C. lavandulifolium* in response to *A. alternata* (Fig. 5D–E). The highly increased transcription levels of *CiWRKY103* caused by 5-AzaC treatment and *A. alternata* inoculation in *C. indicum* further supported the phenotype of resistance to BSD (Fig. 5F). In the cultivar, enhanced resistance was consistently observed in 5-AzaC-treated *C. morifolium*, which was accompanied by a significant upregulation of *CmWRKY103* expression (Fig. S12A–C). These results support the idea that transcriptional activation of *WRKY103*, a typical member of WRKYs, by DNA hypomethylation is likely a common mechanism by which chrysanthemum plants gain resistance to *A. alternata*. These findings also suggest that chemically induced DNA hypomethylation could be a potentially effective method to resist BSD in different chrysanthemum species.

## Discussion

Polyploidy is widely recognized to enhance plant environmental adaptability, yet the underlying mechanisms, particularly concerning biotic stress resistance, remain enigmatic. Studies in rice and citrus have shown that polyploidization can improve salt tolerance through DNA hypomethylation-activated JA pathways (Wang et al. [13]; Song et al. [3]), while analogous epigenetic mechanisms governing fungal disease resistance have remained largely unexplored. Our study bridges this critical gap by establishing a direct connection from polyploidization to CHH hypomethylation-activated WRKYs, and further robust fungal resistance in chrysanthemum. Our work extends the understanding of epigenetic-mediated biotic stress tolerance in polyploids, revealing a potentially conserved strategy for enhanced immunity in plants.

WRKY TFs are well-established essential regulators of plant immunity. In a previous study, *CmWRKY33.1* was found to negatively regulate resistance to BSD in cultivated *C. morifolium* [25]. Phylogenetic analysis revealed that *CmWRKY33.1* is more closely related to *AtWRKY25* and *AtWRKY26* than to classical resistance gene *AtWRKY33* (Fig. S4). In *A. thaliana*, *AtWRKY25* and *AtWRKY26* exhibit expression patterns opposite to that of *AtWRKY33* under heat stress [26], which suggests differences in their regulatory directions and complex regulatory network of WRKYs. Previous studies have shown that *AtWRKY33* and its orthologues play multiple important roles in plant defense. [27]. It is a central hub involved in the response of various plants to *Colletotrichum gloeosporioides*, *A. brassicicola*, and *Botrytis cinerea*. [14, 28, 29]. We found that the hypomethylated and functionally critical *CIWRKY103* is an orthologue of *AtWRKY33* (Fig. S5), which suggests an evolutionary conservation of this defense module. More importantly, our work expands this knowledge by demonstrating how polyploidization epigenetically unlocks the potential of this conserved module.

Polyploidization can lead to both DNA hyper- and hypomethylation in a context-dependent manner in plants [30]. Our study aligns with and refines this idea by illustrating that the response to biotic stress is predominantly orchestrated through locus-specific CHH hypomethylation in polyploids. The high transposable element (TE) content of the chrysanthemum genome (>70%) [31–33], similar to other large plant genomes, provides a landscape rich in targets for CHH methylation, which is maintained by the RdDM pathway [34]. Subsequent pathogen attack then triggers active demethylation or passive loss of methylation at selected targets, such as TE-rich promoter regions of key WRKY genes, a process potentially regulated by DNA demethylases under stress in Arabidopsis [35]. In support of this idea, flanking region of *ClWRKY103* is indeed TE-rich in genome (Fig. 3C). It suggests polyploids with high TE content might exhibit heightened capacity for epigenetic regulation under stresses, a hypothesis that requires further investigation in more plant species.

The conservation of WRKY103-mediated resistance to BSD across diploid, autotetraploid, and even hexaploid chrysanthemum cultivar suggests that the pathway to enhance resistance via DNA hypomethylation is an inherent capacity within the chrysanthemum genus. It indicates that the superior resistance after polyploidization is not merely a consequence of additive effect but can be achieved by epigenetically reprogramming the diploid to mimic the polyploid state. The success of 5-AzaC treatment in inducing resistance across all tested chrysanthemum species validates this concept.

**Figure 6:**
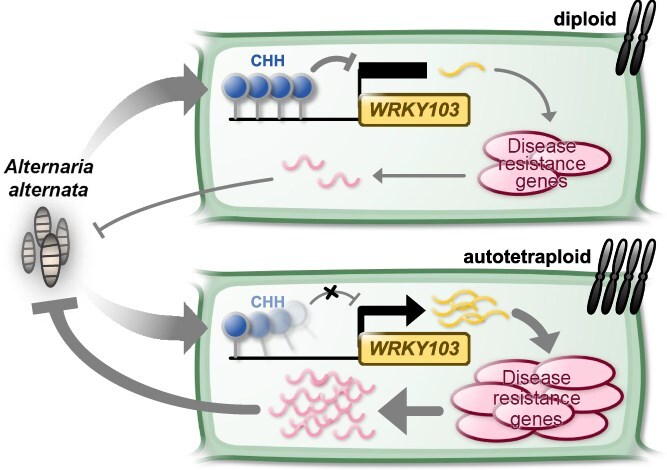
CHH hypomethylation potentiates the transcription of *WRKY103* to increase resistance against *A. alternata* infection in autotetraploid chrysanthemum.

Taken together, the results of this study revealed that CHH hypomethylation potentiates the transcription of WRKYs, particularly *WRKY103*, to increase resistance to *A. alternata* infection in autotetraploid chrysanthemum (Fig. 6). Our work suggests that the ‘genomic shock’ of polyploidization can be harnessed for environmental adaptation through precise epigenetic tuning, specifically by modulating key immune regulators via CHH hypomethylation. By linking established concepts of polyploid epigenetics with the WRKY defense network, our work provides a new paradigm for understanding and engineering disease resistance, particularly fungal pathogen induced disease, in crops.

## Materials and methods

### Plant materials

The materials used in this experiment included diploid *C. lavandulifolium* (2*n* = 2*x* = 18, abbreviated as 2*x Cl*), autotetraploid *C. lavandulifolium* line 1 (2*n* = 4*x* = 36, abbreviated as 4*x Cl*-1), and autotetraploid *C. lavandulifolium* line 2 (2*n* = 4*x* = 36, abbreviated as 4*x Cl*-2); diploid *C. indicum* (2*n* = 2*x* = 18, abbreviated as 2*x Ci*), autotetraploid *C. indicum* line 1 (2*n* = 4*x* = 36, abbreviated as 4*x Ci*-1), and autotetraploid *C. indicum* line 2 (2*n* = 4*x* = 36, abbreviated as 4*x Ci*-2); and cultivar *C. morifolium* ‘Jinba’ (2*n* = 6*x* = 54, abbreviated as 6*x Cm*). All the plant materials were maintained at the Chrysanthemum Germplasm Resource Conservation Center at Nanjing Agricultural University (Nanjing, China) and cultivated under identical agronomic practices in a glass greenhouse. The growth conditions were controlled as follows: day/night temperature regime of 24°C/20°C, 16-h light/8-h dark photoperiod, and 70% relative humidity. After the plants had up to 10 mature leaves, they were used in the experiments.

### *A. alternata* inoculation and sampling

*A. alternata* strains were incubated in 500 ml of potato dextrose water liquid medium (Sigma Aldrich, America) on a shaker at 200 rpm for 24 h [36]. The mycelial pellets (1 cm in diameter) were inoculated onto the abaxial (underside) surface of the fully expanded third and fourth leaves, at both the left and right sides. At 36 h postinoculation, leaves from both treated and control plants were sampled, flash-frozen in liquid nitrogen, and stored at −80°C for subsequent experiments. Each sample included three biological replicates.

### Statistics of leaf disease incidence

The leaf disease incidence (LDI) of inoculated leaves was calculated via the following formula: LDI = (total lesion area/total leaf area) × 100% [37]. The calculation of the lesion area and leaf area was performed via ImageJ (version 1.54f) [38].

### RNA extraction, RNA-seq, and data analysis

Total RNA was isolated from samples at 36 h postinoculation via an RNA extraction kit (Huayueyang Biotechnology, China) following the manufacturer's protocol, with three biological repetitions per material. All the libraries were constructed and sequenced via the DNBSEQ platform at BGI (China) to generate sequence reads.

HISAT2 (version 2.2.1) was used to obtain the expression levels of genes [39]. The reported *C. lavandulifolium* genome was used as a reference genome [31]. The BAM files were sorted via SAMtools (version 1.10), and the gene expression abundance was calculated via StringTie (version 2.2.1) [40, 41]. Finally, gene expression was normalized via TPM (transcripts per million) values via the following formula: TPM = (total number of reads compared with genes/length of genes) × (1 × 10^6^/total number of sequencing reads). The R package DEseq2 (version 1.38.3) was used to analyse the differential gene expression before and after processing [42]. Differentially expressed genes (DEGs) with log2FoldChange values >1 and *P-*values <0.01 were identified by screening.

### Whole-genome bisulfite sequencing and data analysis

Genomic DNA was extracted from samples collected at 36 h postinoculation, with two repetitions per material. Then, it was fragmented to a mean size of 200–350 bp, followed by blunt-ending and 3′ end dA addition. After that, methylated adaptor ligation was performed. Bisulfite conversion was carried out via the EZ DNA Methylation Gold Kit (Zymo Research, America), and a library was constructed. All libraries were sequenced on the DNBSEQ platform at BGI (China) to generate sequence reads.

Bismark (version 0.23.0) was used to map the clean reads onto the *C. lavandulifolium* reference genome [43]. DMRs were identified via Fisher's exact test with an FDR threshold of <0.01 on the basis of 50-bp bin cytosine methylation data. Metagene plots for genes and TEs were generated via deepTools (version 3.5.1) [44]. Differences in methylation levels between the two groups were visualized via R.

### DNA affinity purification sequencing and data analysis

Genomic DNA was extracted from the samples, and size-selected genomic DNA was used to construct an affinity-purified library. Library preparation was performed via the NGS0602-MICH TLX DNA-Seq Kit (MICH SCIENTIFIC, China). After a mixed culture of genomic DNA and protein, the complex was eluted according to the method of Bluescape (China), and sequencing was conducted.

BWA-MEM (version 0.7.17-r1188) was used to map the clean reads onto the reference genome [45]. MACS2 (version 2.2.7.1) was used to call peaks [46], MEME-ChIP (version 5.2.0) was used to analyse and predict motifs in the peak regions [47], and deepTools (version 3.5.1) was used to profile the distribution of reads around transcription start sites (TSSs) [48]. Finally, the gene structure and the motifs in gene promoter regions were visualized with TBtools (version v2.210) [49].

### Phylogenetic analysis

To construct the phylogenetic tree of the WRKY family, multiple sequence alignment was performed using ClustalX (version 2.1) [50], and the aligned file was used for subsequent analysis. Phylogenetic reconstruction was carried out with IQ-TREE (version 2.4.0) [51]. ModelFinder was used to select the best fit substitution model, which was determined to be Q.PLANT+R4. The maximum likelihood (ML) tree was generated under this model, and branch support was assessed with 2000 bootstrap replicates.

### 5-AzaC treatment

5-Azacytidine (5-AzaC) (Sigma, A2385) was dissolved in dimethyl sulfoxide (DMSO) to prepare a 5 mM stock solution, which was stored at −80°C. The stock solution was diluted with sterile water to a working solution of 100 μM and sprayed onto plants at a volume of 2.5 ml per plant, ensuring thorough coverage of the entire plant. Control plants were sprayed with an equivalent volume of an aqueous solution containing 2% DMSO. After treatment, plants were placed in a growth chamber under the following conditions: day/night temperature regime of 24°C/20°C, 16-h light/8-h dark photoperiod, and 70% relative humidity for 3 days. On Day 4, leaves from both treated and control plants were inoculated with *A. alternata*.

### Relative expression analysis by qRT–PCR

The relative expression levels of *WRKY103* in *C. lavandulifolium*, *C. indicum*, and *C. morifolium* were verified via quantitative reverse transcription PCR (qRT–PCR), with three biological replicates and three technical replicates for each sample. Reverse transcription of total RNA (1 mg) was performed via Prime Script™ RT Master Mix (Perfect Real Time; Takara, Japan) following the manufacturer's instructions. Primer Premier 5 design software (Takara, Japan) was used to design gene-specific primer pairs for the selected genes, and *EF1α* was used as an internal reference. The relative expression levels of genes were calculated via the 2^−ΔΔCT^ method [52]. The primers used for qPCR are listed in Table S8.

### *C. lavandulifolium* transformation

The pORE-R4-*ClWRKY103* plasmid was extracted via a plasmid extraction kit (Thermo Fisher Scientific, America). Five hundred nanograms of the purified plasmid was mixed with 50 μl of *Agrobacterium tumefaciens* EHA105 competent cells in a 1.5-ml microcentrifuge tube through gentle pipetting. The mixture was transferred to an electroporation cuvette and subjected to electric pulse treatment at 1800 V via an electroporation system. Following electroporation, the transformed cells were resuspended in 700 μl of liquid YEB medium and incubated at 28°C with agitation at 200 rpm for 1 h. Aliquots (100 μl) of the cell suspension were spread onto YKR selective agar plates and cultured inverted at 28°C for 3 days. Single colonies were subsequently inoculated into 700 μl of liquid YEB medium and cultured at 28°C with shaking at 200 rpm for 8 h prior to colony verification. Positive clones containing the pORE-R4-*ClWRKY103* plant expression vector were identified through diagnostic analysis. Finally, the recombinant plasmid was introduced into *C. lavandulifolium* via Agrobacterium-mediated genetic transformation to obtain overexpressing plants.

## Supplementary Material

Web_Material_uhag050

## Data Availability

All data supporting the findings of this study are available in the article and its supplementary figures and supplementary tables. The gene and protein sequences used in this study were derived from the published *C. lavandulifolium* genome [31]. The raw RNA-seq, WGBS, and DAP-seq data reported in this paper have been deposited in the Genome Sequence Archive (GSA) under project number PRJCA037346 and GSA accession number CRA023867 (https://ngdc.cncb.ac.cn/gsa). All other reasonable requests for data and research materials can be accommodated by reaching out to the authors.
