# Supplementary material for: Polyploidization enhances plant resistance to Alternaria alternata via DNA hypomethylation activated WRKYs
Source: Hortic Res. 2026 Feb 19;13(6):uhag050. doi: 10.1093/hr/uhag050 (PMC13235531; doi:10.1093/hr/uhag050)
Supplement: Web_Material_uhag050 [file web_material_uhag050.zip › 20260119_Supplementary Figures-revised.pdf]

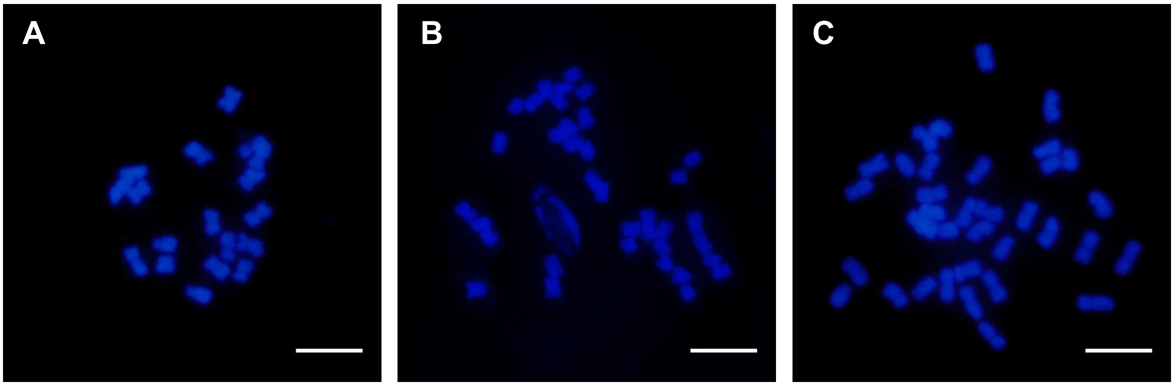

Figure S1 Mitotic metaphase chromosomes of diploid *C. lavandulifolium* and two autotetraploid *C. lavandulifolium* lines.

A Chromosomes of diploid *C. lavandulifolium* (2x *Cl*, 2n=2x=18) stained with DAPI (blue), Scale bar: 10 μm. **B** Chromosomes of tetraploid *C. lavandulifolium* line 1 (4x *Cl*-1, 2n=4x=36) stained with DAPI. **C** Chromosomes of autotetraploid *C. lavandulifolium* line 2 (4x *Cl*-2, 2n=4x=36) stained with DAPI.

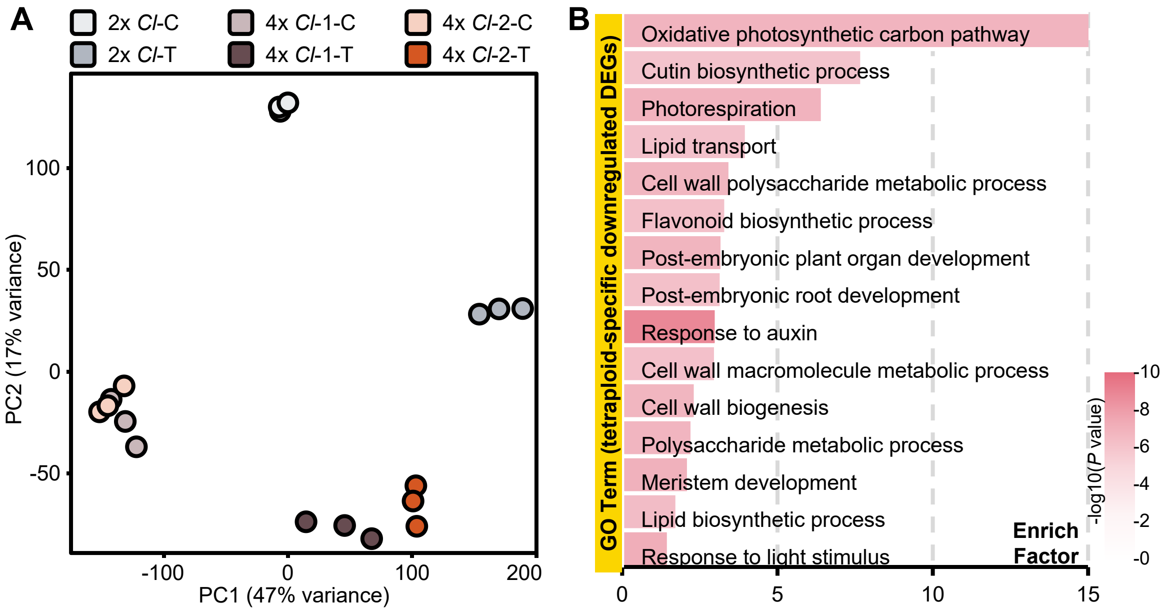

Figure S2 Consistency and enrichment analysis of the transcriptomic data.

A PCA plot of gene expression levels in diploid and autotetraploid *C. lavandulifolium*. 2x *Cl*: diploid *C. lavandulifolium*; 4x *Cl*-1: autotetraploid *C. lavandulifolium* line 1; 4x *Cl*-2: autotetraploid *C. lavandulifolium* line 2. **B** Gene ontology (GO) enrichment analysis of the tetraploid-specific downregulated DEGs.

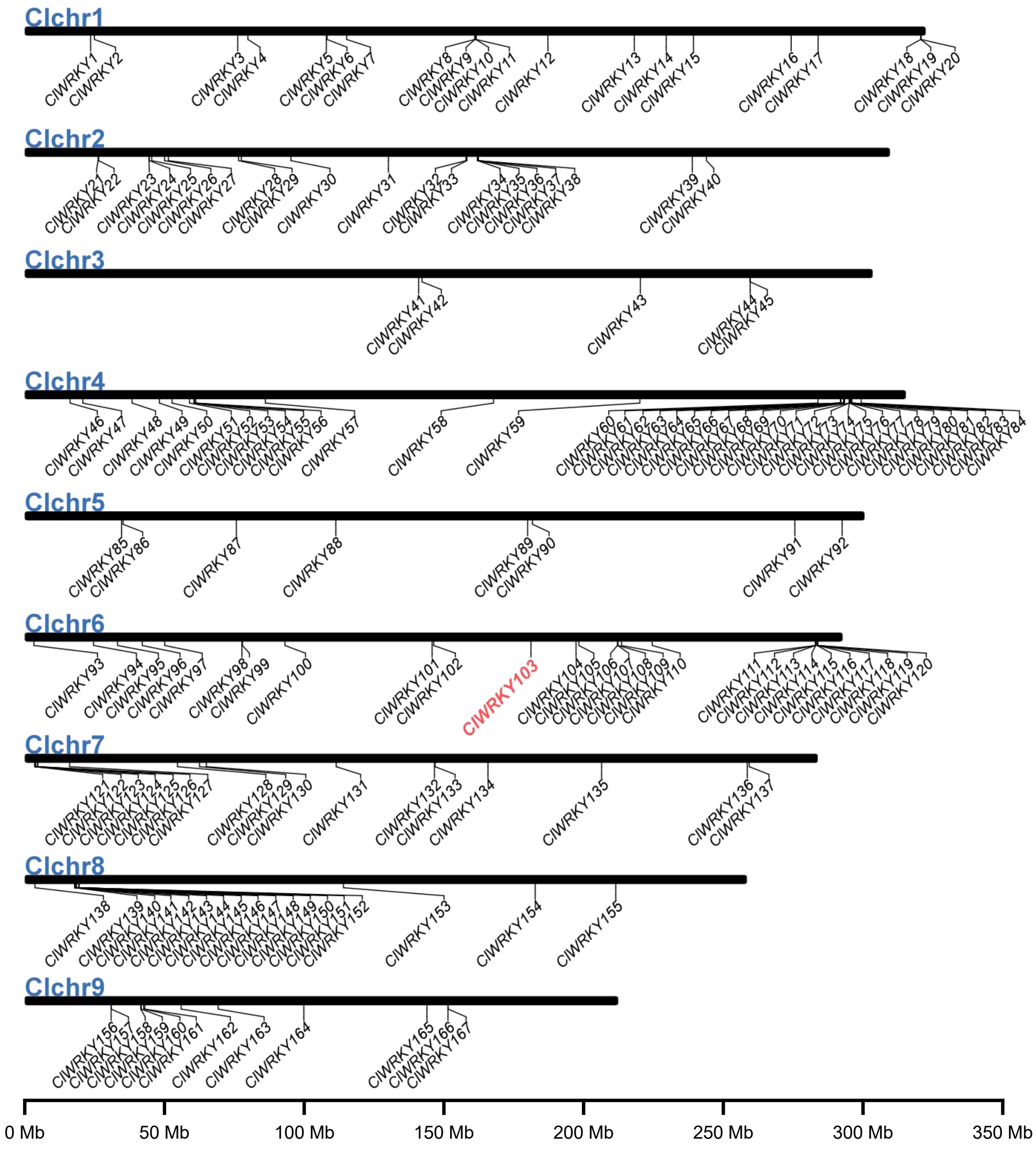

Figure S3 The position of WRKY family genes on the genome of *C. lavandulifolium*.

The 167 identified WRKY genes were mapped onto the *C. lavandulifolium* chromosomes (Clchr1 to Clchr9). The scale bar bar at the bottom indicates the physical distance in Mb (Megabases). The gene names are marked with lines extending from their positions.

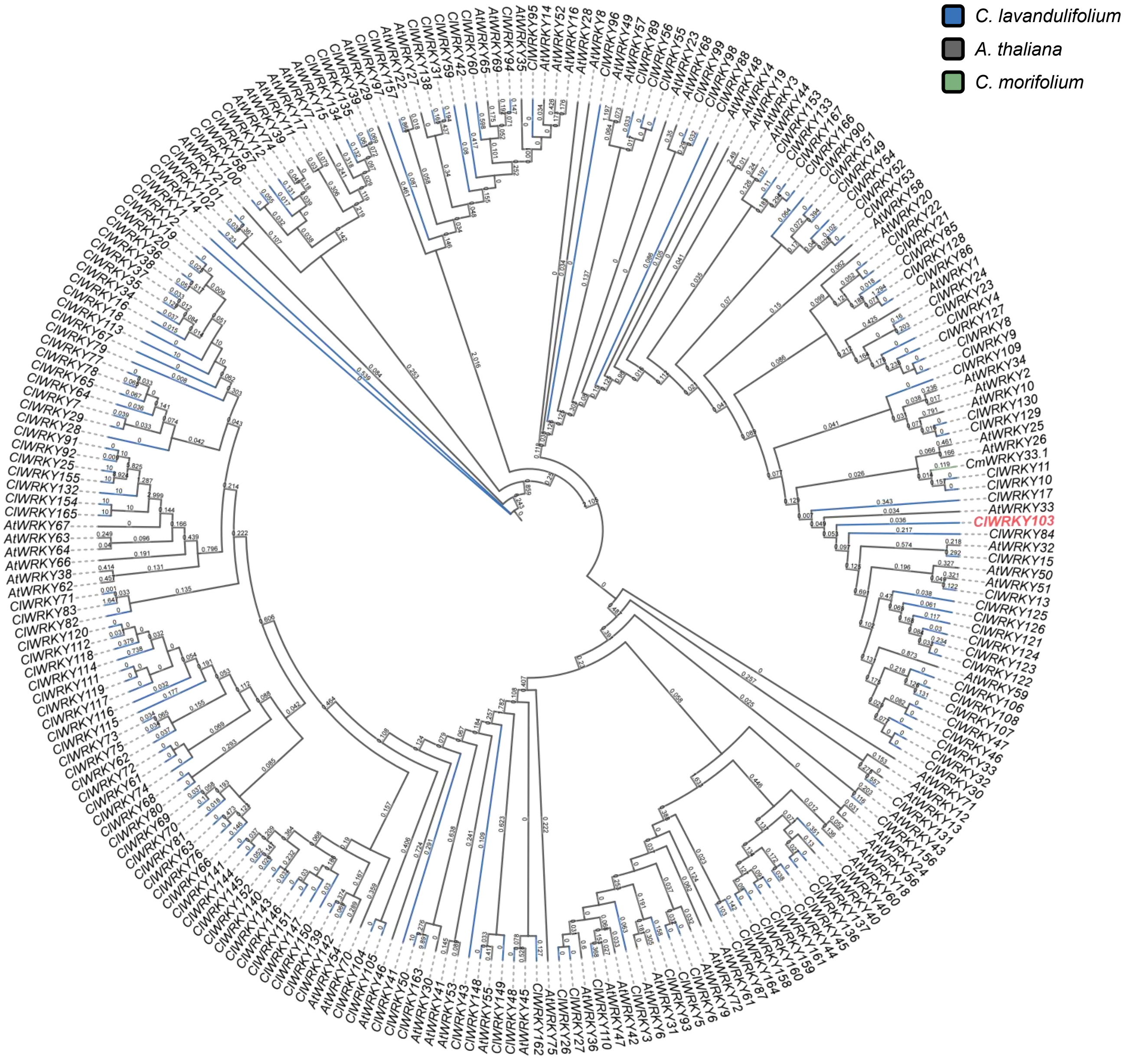

Figure S4 Evolutionary tree of WRKY family genes.

Blue represents *C. lavandulifolium* WRKY genes (*ClWRKY*), Grey represents *A. thaliana* WRKY genes (*AtWRKY*), and Green represents *C. morifolium* WRKY gene (*CmWRKY33.1*). Branch lengths were ignored in the tree layout, with each branch length labeled on its branch.

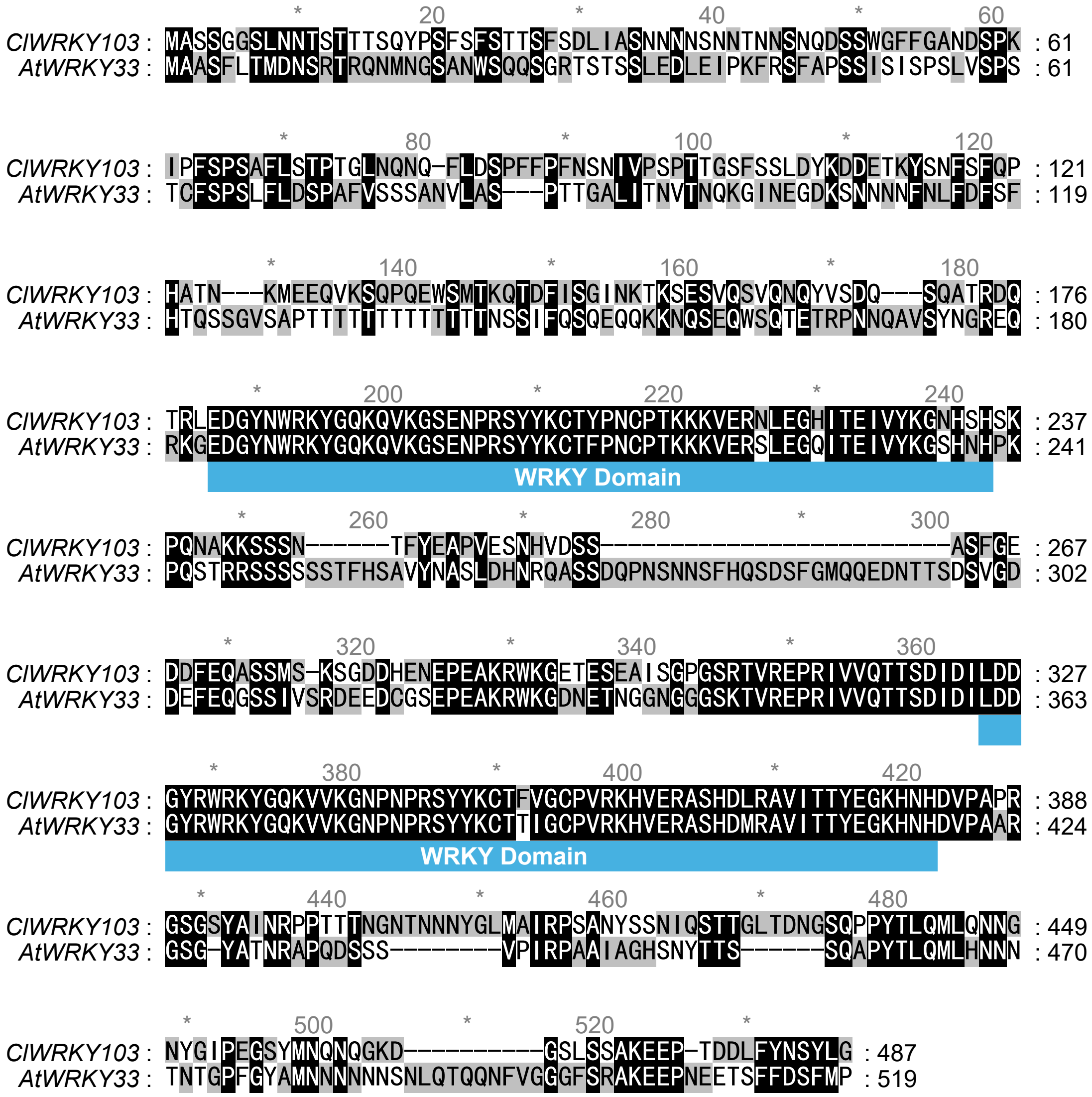

Figure S5 Sequence alignment and domain architecture of ClWRKY103 and AtWRKY33 proteins.

Blue rectangles indicate the positions of the WRKY domains. Numbers to the right of the proteins denote the positions within the original sequences, while numbers above the proteins represent the aligned positions of the sequences.

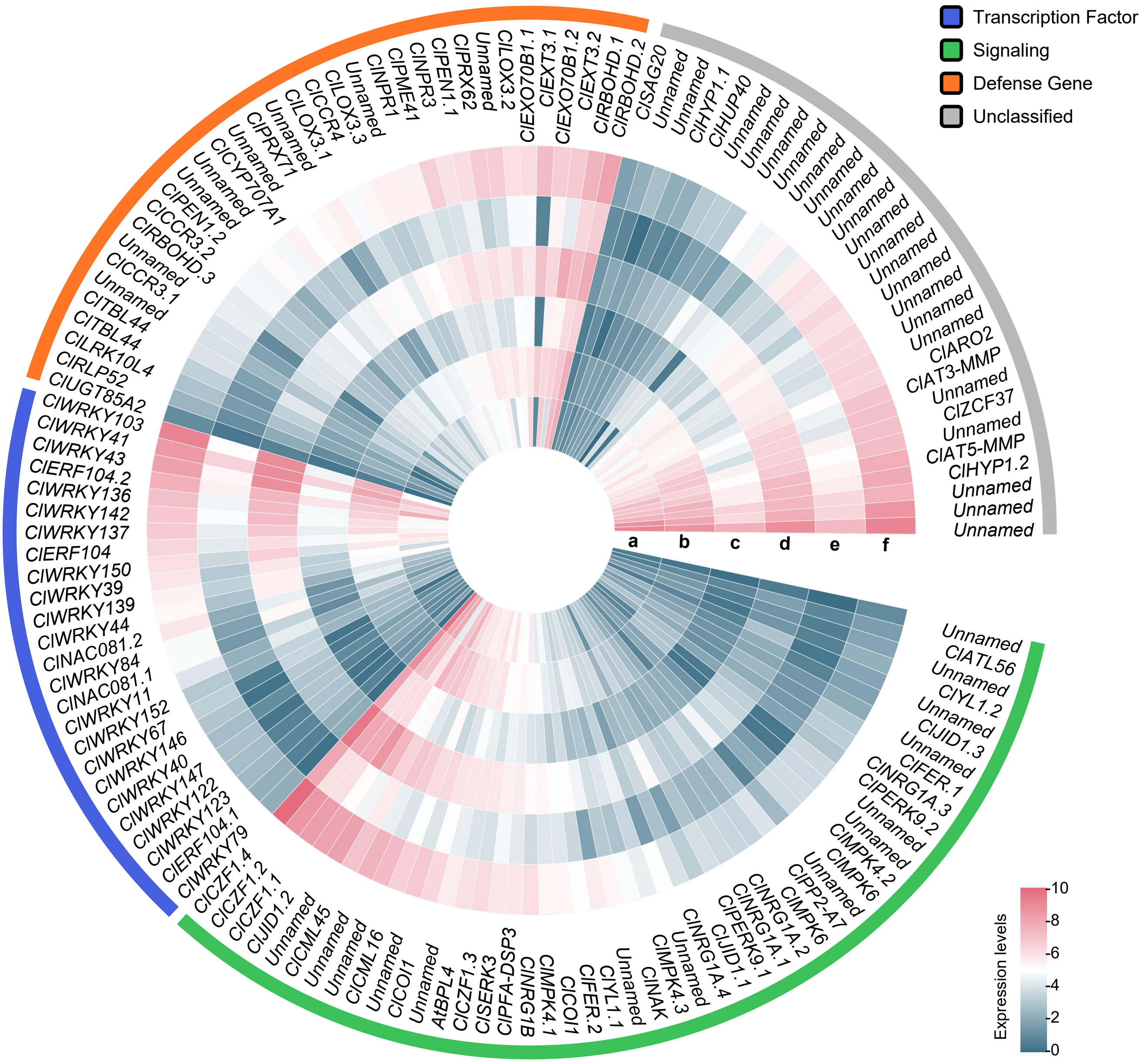

Figure S6 Heatmap of gene expression in response to fungus.

'Response to fungus' (GO:0009620) was identified as a significant term through GO enrichment analysis in the set of tetraploid-specific upregulated genes. **A** Control group of diploid *C. lavandulifolium*. **B** Inoculation treatment group of diploid *C. lavandulifolium*. **C** Control group of autotetraploid *C. lavandulifolium* line 1. **D** Inoculation treatment group of autotetraploid *C. lavandulifolium* line 1. **E** Control group of autotetraploid *C. lavandulifolium* line 2. **F** Inoculation treatment group of autotetraploid *C. lavandulifolium* line 2. Blue: Transcription factor genes, Green: Signaling genes, Orange: Defense genes, Gray: Unclassified genes. Color bar represent normalized gene expression levels (TPM).

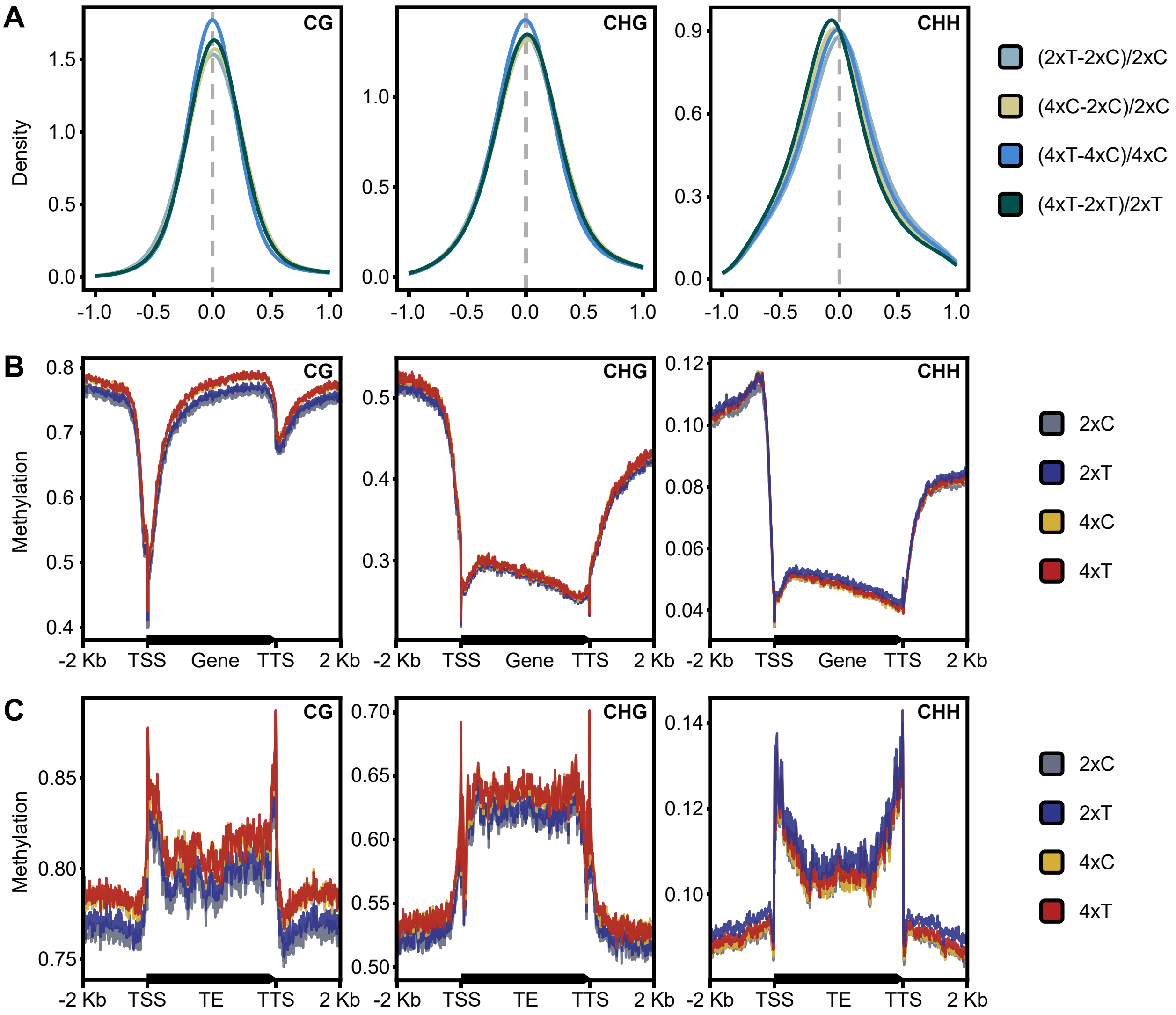

Figure S7 DNA methylation status of diploid and autotetraploid *C. lavandulifolium* after *A. alternata* inoculation.

A DNA methylation changes after autopolyploidization and *A. alternata* inoculation. The Y-axis represents probability density. C: Control group, T: Treatment group inoculated with *A. alternata*, Light blue: Comparison of diploid *C. lavandulifolium* before and after inoculation, Yellow: Comparison of *C. lavandulifolium* in control group before and after autopolyploidization, Sky blue: Comparison of autotetraploid *C. lavandulifolium* before and after inoculation, Green: Comparison of *C. lavandulifolium* in treatment group before and after autopolyploidization. **B** Distributions of methylation in coding genes. The Y-axis represents the methylation ratio, ranging from 0 to 1 (0% to 100%). 2x: diploid *C. lavandulifolium*; 4x: autotetraploid *C. lavandulifolium* line 1, Gray: Control group of diploid *C. lavandulifolium*, Blue: Inoculation treatment group of diploid *C. lavandulifolium*, Yellow: Control group of autotetraploid *C. lavandulifolium*, Red: Inoculation treatment group of autotetraploid *C. lavandulifolium*. **C** Distributions of methylation in TEs. The Y-axis represents the methylation ratio, ranging from 0 to 1 (0% to 100%).

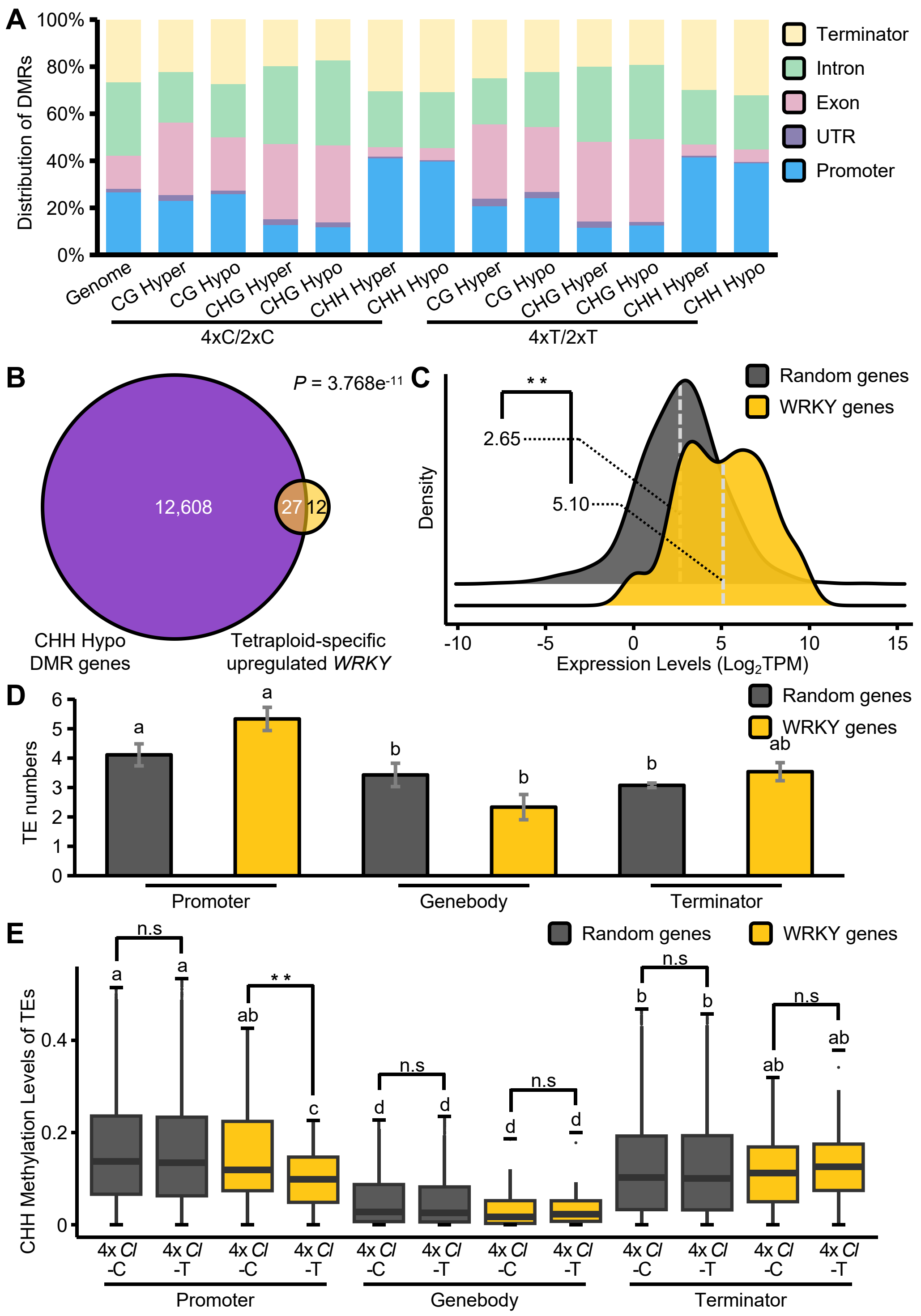

Figure S8 DNA methylation was associated with the expression patterns of WRKY genes.

A Distribution of DMRs in gene-related regions of the genome. Blue: Promoter region, Purple: Untranslated Region, Pink: Exon region, Green: Intron region, Yellow: Terminator region. **B** Venn analysis of CHH Hypo DMR genes and tetraploid-specific upregulated WRKY genes. Purple: CHH Hypo DMR genes, Golden: WRKY genes. Significance of differences: Hypergeometric test. **C** Differences in expression level. Grey: 1000 randomly selected genes, Golden: Tetraploid-specific upregulated WRKY genes (**: *P* < 0.01, one-way ANOVA). **D** Differences in TE numbers. Grey: 1000 randomly selected genes, Golden: Tetraploid-specific upregulated WRKY genes. Significance of differences: Different lowercase letters indicate significant differences between groups (one-way ANOVA). **E** Differences in CHH Methylation Levels of TEs. Grey: 1000 randomly selected genes, Golden: Tetraploid-specific upregulated WRKY genes. Significance of differences: Different lowercase letters indicate significant differences between groups (**: *P* < 0.01, one-way ANOVA).

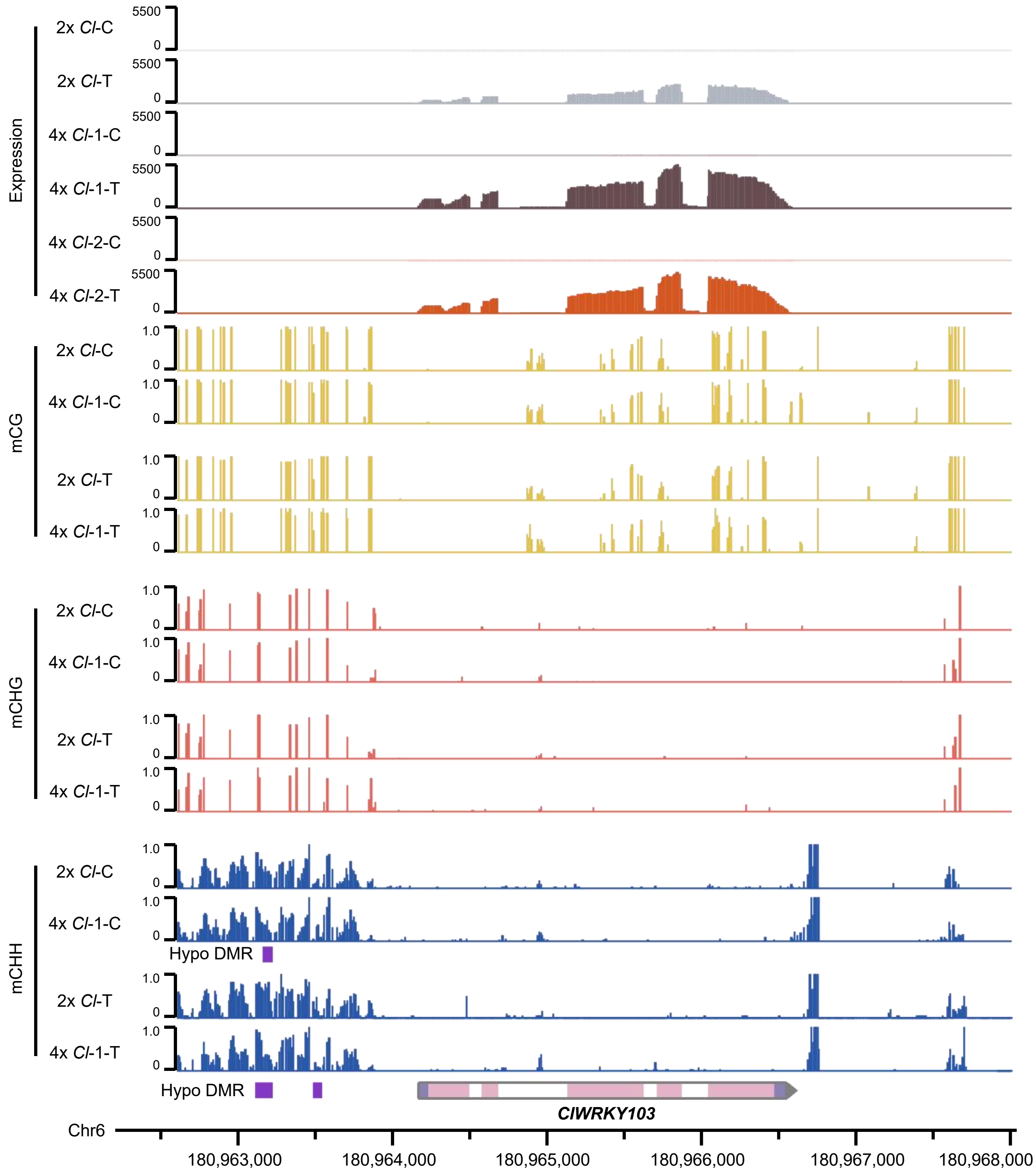

Figure S9 Expression and DNA methylation levels in *ClWRKY103* regulatory and genic regions.

Panel Expression displays 6 tracks of gene expression levels for different samples, with the Y-axis from 0 to 5,500 represents the expression levels in TPM. Panel mCG, mCHG and mCHH displays tracks of DNA methylation levels for different samples, with the Y-axis from 0 to 1.0 represents the methylation ratio from 0% to 100%. 2x *Cl*: diploid *C. lavandulifolium*, 4x *Cl*-1: autotetraploid *C. lavandulifolium* line 1, 4x *Cl*-2: autotetraploid *C. lavandulifolium* line 2, C: Control group; T: Treatment group inoculated with *A. alternata*; Pink: Exon; Light purple: Untranslated Region; Yellow: CG methylation; Red: CHG methylation; Blue: CHH methylation; Purple: Hypomethylated DMR.

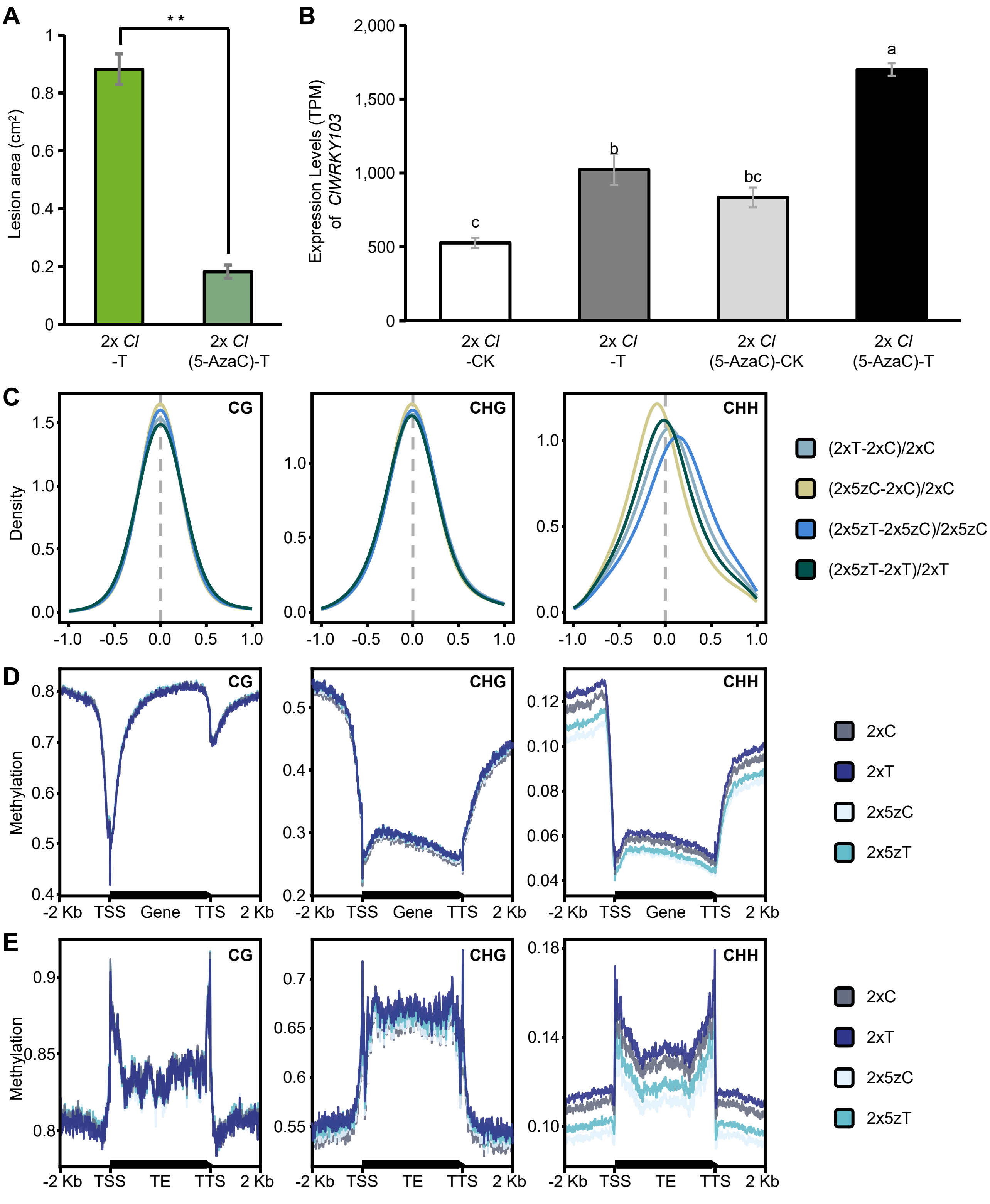

Figure S10 5-AzaC treatment increased BSD resistance in *C. lavandulifolium.*

A Statistical analysis of leaf lesion area. 2x *Cl*: diploid *C. lavandulifolium,* C: Control group, T: Treatment group inoculated with *A. alternata*, Error bars represent standard error (SE, n=10), Significance of differences: **: $P < 0.01$ (one-way ANOVA). **B** The expression levels of *ClWRKY103* in the control group versus the *A. alternata* inoculated treatment group under 2x *Cl* and 2x *Cl* (5-AzaC) conditions. **C** Global methylation changes after 5-AzaC treatment and *A. alternata* inoculation. The Y-axis represents probability density. Diploid *C. lavandulifolium* with (2xT) or without (2xC) *A. alternata* inoculation, 5-AzaC-sprayed diploid *C. lavandulifolium* with (2x5zT) or without (2x5zC) *A. alternata* inoculation. **D** Distributions of methylation in coding genes. The Y-axis represents the methylation ratio, ranging from 0 to 1 (0% to 100%). Gray: Control group of diploid *C. lavandulifolium*, Blue: Inoculation treatment group of diploid *C. lavandulifolium*, Light blue: Control group of 5-AzaC-sprayed diploid *C. lavandulifolium*, Cyan: Inoculation treatment group of 5-AzaC-sprayed diploid *C. lavandulifolium*. **E** Distributions of methylation in TEs. The Y-axis represents the methylation ratio, ranging from 0 to 1 (0% to 100%).

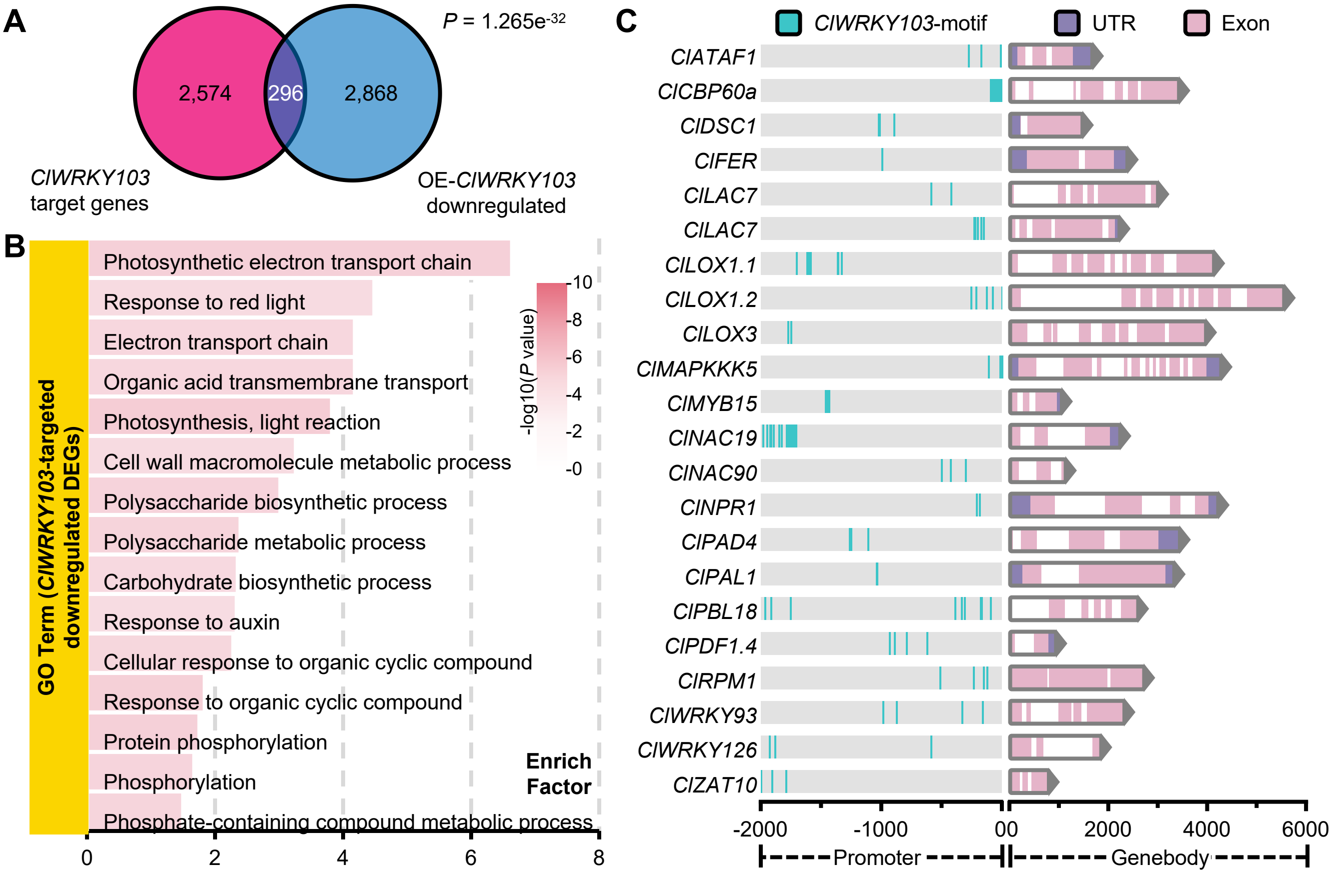

Figure S11 Transcriptional responses of *ClWRKY103* downstream genes.

A Venn plot of *ClWRKY103* target genes and downregulated DEGs in OE-*ClWRKY103.* Significance of differences: Hypergeometric test. **B** GO enrichment analysis of downregulated DEGs in *ClWRKY103* target genes. **C** *ClWRKY103* target genes and their promoter *ClWRKY103*-motif binding sites. Cyan: *ClWKRY103*-motif, Pink: Exon, Purple: Untranslated Region.

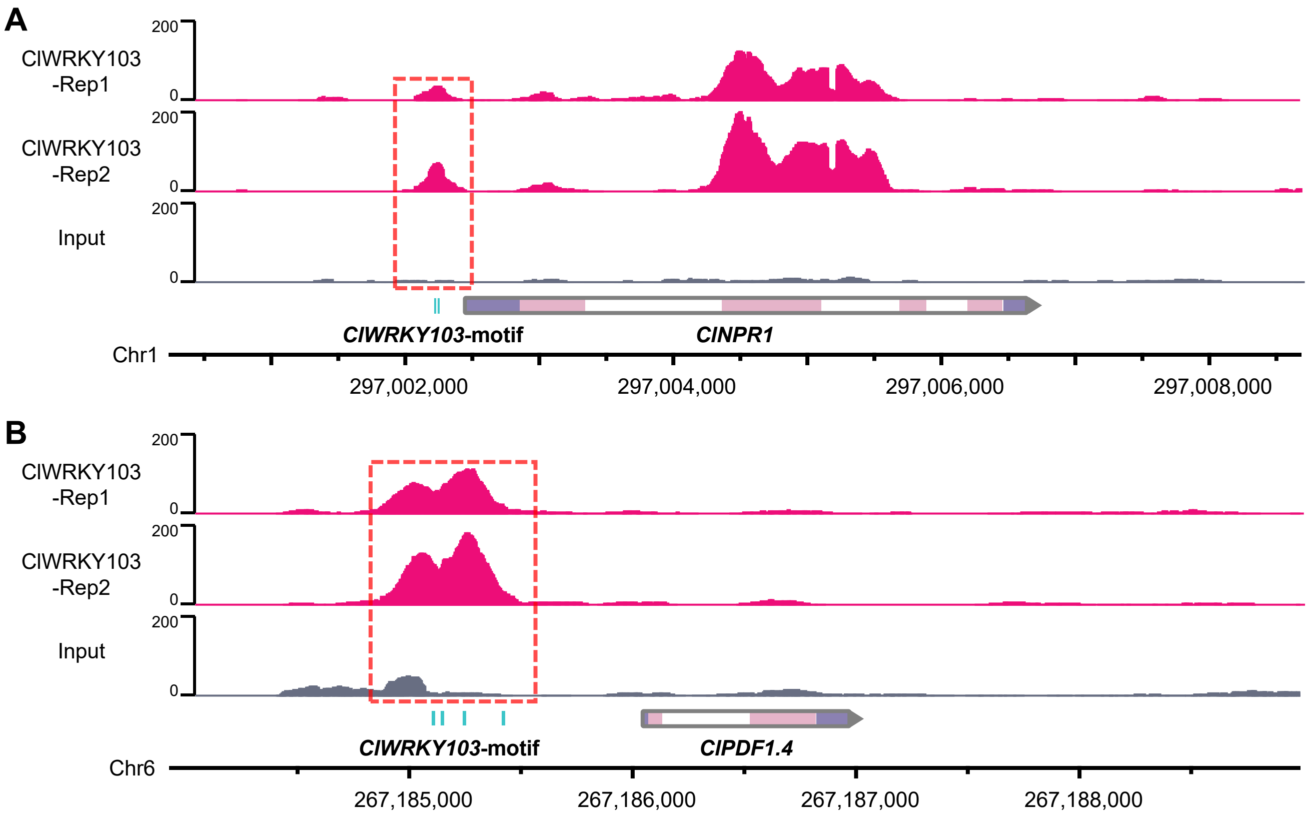

Figure S12 DAP-seq signal enrichment of *ClWRKY103* target genes.

A Genome browser snapshots showing DAP-seq signal enrichment at the *ClNPR1* locus. Panel displays 3 tracks of normalized read coverage for ClWRKY103 libraries and the input library as a control, with the Y-axis from 0 to 200 represents the normalized read coverage. ClWRKY103-Rep1: ClWRKY103 DAP-seq repeat1, ClWRKY103-Rep2: ClWRKY103 DAP-seq repeat2, Input: input DNA library. Pink: Exon; Light purple: Untranslated Region; Cyan: *ClWKRY103*-motif. **B** Genome browser snapshots showing DAP-seq signal enrichment at the *ClPDF1.4* locus. Panel displays 3 tracks of normalized read coverage for ClWRKY103 libraries and the input library as a control, with the Y-axis from 0 to 200 represents the normalized read coverage. ClWRKY103-Rep1: ClWRKY103 DAP-seq repeat1, ClWRKY103-Rep2: ClWRKY103 DAP-seq repeat2, Input: input DNA library. Pink: Exon; Light purple: Untranslated Region; Cyan: *ClWKRY103*-motif.

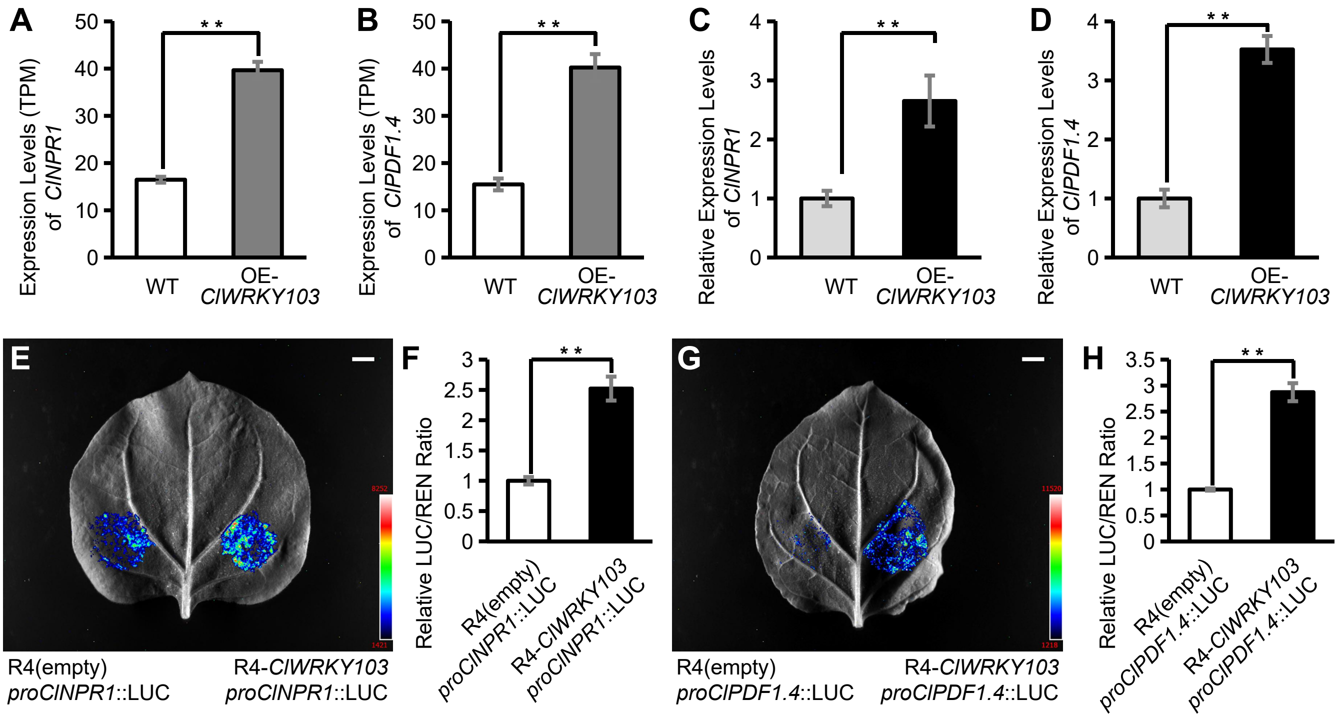

Figure S13 Dual-LUC and expression analyses of *ClWRKY103* target genes.

A Expression levels of *ClNPR1* in the WT and OE-*ClWRKY103*. Significance of differences: **: *P* < 0.01 (DEseq2). **B** Expression levels of *ClPDF1.4* in the WT and OE-*ClWRKY103*. Significance of differences: **: *P* < 0.01 (DEseq2). **C** The relative expression levels of *ClNPR1* in the WT and OE-*ClWRKY103*. Significance of differences: **: *P* < 0.01 (Student's *t*-test). **D** The relative expression levels of *ClPDF1.4* in the WT and OE-*ClWRKY103*. Significance of differences: **: *P* < 0.01 (Student's *t*-test). **E** LUC imaging assays for *ClWRKY103* and the promoter of *ClNPR1*. Scale bar: 1 cm. **F** Statistical analysis of dual LUC assay for *ClWRKY103* and the promoter of *ClNPR1*. Error bars represent the standard error (SE, n=3). Significance of differences: **: *P* < 0.01 (student t-test). **G** LUC imaging assays for *ClWRKY103* and the promoter of *ClPDF1.4*. **H** Statistical analysis of dual LUC assay for *ClWRKY103* and the promoter of *ClPDF1.4*. Error bars represent the standard error (SE, n=3). Significance of differences: **: *P* < 0.01 (Student's *t*-test).

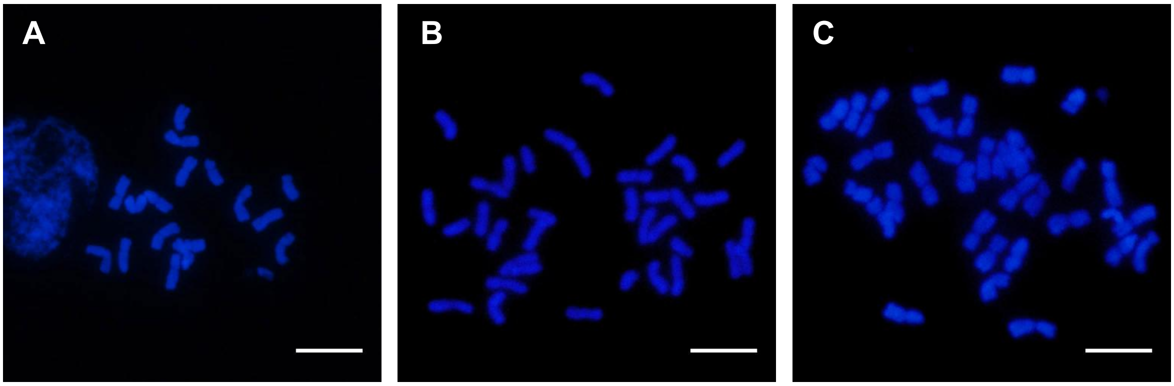

Figure S14 Mitotic metaphase chromosomes of diploid *C. indicum* and two autotetraploid *C. indicum* lines.

A Chromosomes of diploid *C. indicum* (2x *Ci*, 2n=2x=18) stained with DAPI (blue), Scale bar: 10 μm. **B** Chromosomes of tetraploid *C. indicum* line 1 (4x *Ci*-1, 2n=4x=36) stained with DAPI. **C** Chromosomes of autotetraploid *C. indicum* line 2 (4x *Ci*-2, 2n=4x=36) stained with DAPI.

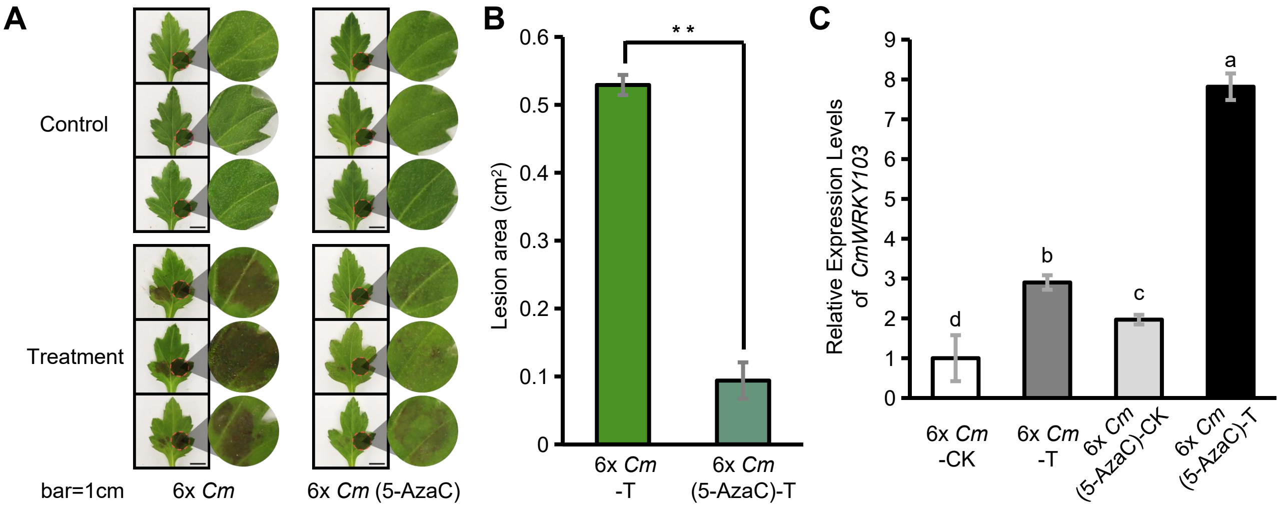

Figure S15 5-AzaC treatment enhanced resistance to *A. alternata* in *C. morifolium*.

A The phenotypic response of 6x *Cm* and 5-AzaC-sprayed leaves to *A. alternata* inoculation. 6x *Cm*: hexaploid *C. morifolium* 'Jinba', Scale bar: 1 cm. **B** Statistical analysis of leaf lesion area. Error bars represent standard error (SE, n=10), Significance of differences: **: $P < 0.01$ (one-way ANOVA). **C** The relative expression levels of *CmWRKY103* in the control group versus the *A. alternata* inoculated treatment group under 6x *Cm* and 6x *Cm* (5-AzaC) conditions. Significance of differences: different lowercase letters indicate significant differences between groups ($P < 0.01$, one-way ANOVA).
